# Prospective study looking at CT staging for metastases in early breast cancer

**DOI:** 10.1186/bcr3801

**Published:** 2015-11-05

**Authors:** Michelle McMahon, Nisha Sharma, David Dodwell

**Affiliations:** 1 The Leeds Teaching Hospitals, NHS Trust, Leeds, UK

## Introduction

Practice is variable nationally with no agreed guidelines for performing CT staging of asymptomatic patients with a new diagnosis of breast cancer. We have devised a new proforma for performing staging CT in asymptomatic women with high-risk early breast cancer. In our unit, 600 cancers are diagnosed/year.

## Methods

Prospective audit identifying patients eligible for CT staging based on our proforma over a 12-month period were identified at the breast cancer MDT. A staging scan of the chest, abdomen and pelvis was performed. CT results and clinic letters were reviewed. Criteria: asymptomatic patients diagnosed with new breast cancer requiring staging (T4, inflammatory breast cancer or tumour which extends into the chest wall, skin, or both, fixed nodal disease, arm oedema, nodal disease in SCF; T3, tumour >50 mm clinically, radiologically or pathologically; ≥4 positive nodes at surgery; part of clinical trial involvement or extensive residual disease at surgery after NACT).

## Results

Forty patients were referred for a CT staging, four patients did not proceed. Indications: 26 (65 %) had four or more metastatic nodes, six (15 %) T3, eight (20 %) T4. 20/36 (56 %) had no evidence of metastatic disease; 8/36 (22 %) had definite metastases identified (four, >4 nodes, three T4 and one T3); 8/36 (22 %) had indeterminate findings. In three cases the diagnosis of metastatic disease contributed to the decision not to proceed with surgery. No negative impact on treatment was reported in the indeterminate cases.

## Conclusion

The new proforma for guiding staging CT scans has reduced the number of overall scans performed with a relatively high pick-up rate of 22 %.

